# Research progress on discharge readiness service practices for patients undergoing endometrial cancer surgery

**DOI:** 10.3389/fonc.2025.1664064

**Published:** 2026-01-15

**Authors:** Xia Chen, Peijuan Tang, Chunjing Ma, Mei Su, Jiaxin Sun, Wenzhong Chang, Yaru Li, Yajuan Cui, Yanting Wang, Yuchong Hu, Jia Wang, Yinyi Wei

**Affiliations:** 1 Department of Gynaecology, Inner Mongolia People’s Hospital, Hohhot, Inner Mongolia, China; 2 Department of Gynaecology, Qilu Hospital of Shandong University, Jinan, Shandong, China; 3 Department of Clinical Medical Research Center, Affiliated Hospital of Inner Mongolia Medical University, Hohhot, Inner Mongolia, China; 4 Department of Nursing, Inner Mongolia People’s Hospital, Hohhot, Inner Mongolia, China; 5 Department of Nursing, Ordos Traditional Chinese Medicine Hospital, Ordos, Inner Mongolia, China; 6 School of Nursing, Inner Mongolia Medical University, Hohhot, Inner Mongolia, China

**Keywords:** discharge readiness service, endometrial cancer, narrative review, surgical patients, transitional care

## Abstract

**Objective:**

This narrative review aims to synthesize and evaluate existing evidence and practices regarding discharge readiness services for patients undergoing endometrial cancer (EC) surgery, with the goal of providing a reference for clinical practice and future research.

**Background:**

The incidence of EC is rising globally. With the widespread adoption of enhanced recovery after surgery (ERAS) protocols and minimally invasive techniques, hospital stays are shortening, making effective discharge planning crucial for ensuring a safe transition to home and preventing readmissions.

**Methods:**

As a narrative review, this article involved a comprehensive but non-systematic examination of literature from PubMed, Web of Science, CNKI, and Wanfang databases, focusing on key components of discharge readiness. These components include assessment tools, service content development, implementation processes, and outcome evaluation. The synthesis prioritized recent evidence and internationally recognized guidelines.

**Results:**

The review identifies that while generic discharge assessment tools are valuable, they require adaptation to address the specific needs of EC patients (e.g., lymphedema risk, sexual health). Effective service implementation relies on a systematic interdisciplinary collaboration model and nurse-led, personalized education (e.g., using the teach-back method). The integration of digital health platforms shows promise for supporting post-discharge care. Outcome evaluation should encompass both clinical indicators (e.g., 30-day readmission rates) and patient-reported outcomes (e.g., using the Health Education Impact Questionnaire). Current challenges include a lack of standardized pathways and fragmented community resources.

**Conclusion:**

Discharge readiness is a critical determinant of recovery quality for EC surgical patients. This review consolidates core components and processes into a practical framework, highlighting the need for multidisciplinary collaboration, patient-centered education, and technology integration. Future efforts should focus on developing standardized, culturally adapted pathways and conducting robust comparative effectiveness research to establish high-quality, evidence-based service systems.

**Relevance to clinical practice:**

This study focuses on the development of core components and processes for systematic discharge readiness services for postoperative endometrial cancer patients, as well as the identification of practice challenges. The findings advocate for the clinical adoption of standardized frameworks and the enhancement of implementation capacity to optimize discharge transitions, patient recovery, and continuity of care.

## Highlights

The identification of key components and processes for systematic discharge readiness services in postoperative endometrial cancer patients is crucial. Establishing a standardized service framework is fundamental for optimizing discharge transitions and ensuring continuity of care. Moreover, enhancing clinical implementation capacity significantly amplifies the framework’s positive impact on patient recovery outcomes.

## 1 Introduction

Endometrial cancer (EC) remains one of the most common gynecologic malignancies worldwide, with a steadily increasing incidence noted in both global statistics and data from China (1, 2). Surgery remains the primary treatment modality for EC, as recommended by the latest guidelines from the European Society of Gynaecological Oncology(ESGO) (1), often complemented by radiotherapy, chemotherapy, or hormone therapy (3). In recent years, advancements in surgical techniques, particularly the adoption of minimally invasive and robot-assisted procedures, coupled with the implementation of Enhanced Recovery After Surgery (ERAS) protocols, have significantly shortened hospital lengths of stay (4, 5). While this trend enhances healthcare efficiency and is often preferred by patients, it concurrently compresses the timeframe available for postoperative preparation and education, potentially elevating the risk of adverse events after discharge (6).

Postoperative recovery from EC surgery is not without challenges. Patients frequently contend with issues such as pain, urinary retention, lower limb lymphedema, sexual dysfunction, and psychological distress, all of which can profoundly impact their quality of life and long-term recovery (7–9). Therefore, ensuring that patients are adequately prepared for discharge—a concept known as discharge readiness—is paramount. Adequate discharge readiness facilitates a safe and effective transition from hospital to home, helps to reduce preventable readmissions, and supports optimal patient outcomes (10, 11).

The concept of discharge readiness has evolved considerably since its inception. Initially focused on physiological stability, it now encompasses a multidimensional construct including the patient’s knowledge, self-care abilities, psychosocial status, and the adequacy of anticipated support at home (12, 13). Theoretical frameworks, such as the one developed by Galvin et al., posit discharge readiness as the culmination of processes involving assessment, education, and coordination (14). In practice, nursing-led discharge readiness services are pivotal, integrating needs assessment, patient education, and care coordination to bridge the gap between hospital and home (15, 16).

Despite its recognized importance, the standardization of discharge readiness services for EC patients, especially within the Chinese healthcare context, remains in its developmental stages. Challenges persist at multiple levels: a lack of detailed, EC-specific implementation guidelines; inefficient coordination mechanisms between hospitals and community care; and sociocultural barriers that may hinder open communication about sensitive topics like sexual health (17, 18). Conversely, discharge readiness services, particularly for EC patients, are still in an exploratory and developmental stage (19), encountering several challenges. Consequently, there is a pressing need to synthesize existing evidence and international experiences to inform the development of structured, effective, and culturally sensitive discharge readiness services for this patient population.

This narrative review seeks to address this gap by systematically describing the core components of discharge readiness services for EC surgical patients. It will synthesize evidence on assessment tools, the construction of service content, implementation processes, and outcome evaluation. By integrating these elements and proposing an implementation flowchart, this review aims to offer clinicians a practical, evidence-informed reference to enhance the discharge transition experience, improve patient recovery, and lay the groundwork for establishing standardized service pathways in the future.

## 2 Literature search and screening strategy

This study adopts a “narrative review” approach, aiming to conduct a descriptive synthesis and interpretation of the existing evidence, practical models, and theoretical frameworks in the field of discharge readiness services for patients undergoing EC surgery. To ensure the breadth and representativeness of the content, an extensive literature search and screening were performed; however, the full procedures of a systematic review (e.g., exhaustive search, double independent screening, rigorous risk of bias assessment, or quantitative synthesis) were not followed.

### 2.1 Literature sources and screening rationale

To construct the content of this review, the authors searched four databases: PubMed, Web of Science, China National Knowledge Infrastructure (CNKI), and Wanfang Data Knowledge Service Platform. The search timeframe primarily focused on from January 2010 to December 2024. The core rationale for literature screening was to identify studies highly relevant to the key components of discharge readiness services for post-EC surgical patients, including assessment tools, service content, implementation processes, and outcome evaluation. During the search, Chinese and English subject terms were used comprehensively, such as “endometrial cancer”, “discharge planning”, “transitional care”, and “discharge readiness”. Literature inclusion was based on the “representativeness and contribution of the studies to the purpose of this review”, rather than conducting a systematic search of all relevant literature.

### 2.2 Considerations for literature inclusion

In the literature screening process, the following aspects were primarily considered: ① Study population: Patients who underwent surgical treatment for endometrial cancer; ② Intervention/Topic: Studies focusing on the assessment, implementation, or evaluation of discharge readiness services and transitional care; ③ Study type: Priority was given to randomized controlled trials (RCTs), cohort studies, qualitative studies, systematic reviews, clinical guidelines, and expert consensuses. Additionally, other literature that could provide important insights was included as appropriate; ④ Language: Literature published in Chinese or English. The exclusion criteria included: non-research literature (e.g., commentaries, editorials), studies whose population did not consist of post-EC surgical patients, or literature whose content was irrelevant to the core components of discharge readiness services.

## 3 Discharge readiness assessment tools for EC surgery patients

Postoperative recovery in patients undergoing EC surgery exhibits significant variability, necessitating a tailored approach to care. The discharge process represents a critical transition point in the continuum of care, bridging the in-hospital phase and post-discharge recovery. To ensure continuity of care, the utilization of comprehensive discharge readiness assessment tools is indispensable (13). Existing tools, while addressing fundamental dimensions such as health status, psychosocial support, and self-management capabilities, are predominantly generic. These tools are beneficial for general patient recovery but lack specificity to meet the unique needs of EC patients. EC patients present distinct challenges due to the unique characteristics of the disease, treatment modalities (e.g., extent of surgery, adjuvant therapy), and specific rehabilitation requirements, such as the risk of lymphedema, sexual function, and fertility counseling. Current discharge readiness tools require targeted adaptation to effectively address these specialized needs. A critical review of their psychometric properties highlights important gaps in validity and applicability. While several instruments demonstrate varying degrees of psychometric robustness (**Table 1**), none have been comprehensively validated in EC populations. Consequently, even as ERAS protocols are widely implemented in gynecologic oncology settings (20), the lack of discharge assessment frameworks tailored for EC patients remains a critical gap in ensuring personalized continuity of care.

**Table 1:** Summary of discharge readiness assessment tools.

Tool name	Time of application	Validated population/context	Validation status in EC	Core dimensions/items	Assessment content	Strengths, limitations, and applicability for EC surgical patients
A. Tools validated or applied in oncological populations (with emphasis on EC)						
Caregiver preparedness scale, CPS) (66, 67)	Prior to Discharge	Cancer patients (68)	Non-EC patients	-/8	Physiological Preparedness, Emotional Needs, Service Planning, Caregiver Burden, Comfort Care, Emergency Coping/Management, Access to Health Information/Resources, Overall Preparedness	**Strength:** Directly assesses cancer caregivers, covering multiple dimensions; **Limitation:** Not self-reported by patients and not optimized for the specific needs of gynecological cancer caregivers, and it lacks items related to sexual function and lymphedema; **Applicability:** Core tool for evaluating caregiver preparedness in EC patients, recommended for use.
Quality of Discharge Teaching Scale, ODTS) (69)	Prior to Discharge	Cancer patients (70)	Non-EC patients	2/6	Perceived Discharge Needs, Pre-discharge Education Received, Guidance Skills/Effectiveness	**Strength:** Focuses on discharge education quality (not just quantity), critical for cancer patients' symptom management; **Limitation:** Lacks EC-specific educational content and items related to sexual function and lymphedema; **Applicability:** High-quality tool for evaluating and improving discharge education for EC patients.
Blaylock Risk Assessment Screening Score,BRASS (71)	At Admission	Lung cancer patients (72)	Non-EC patients	-/10	Age, Social Support, Functional Status, Cognition, Behavioral Patterns, Mobility, Sensory Impairment, Prior Hospitalizations/ED Visits, Current Diagnoses, Medication Classes	**Strength:** Comprehensively covers multiple classic risk factors; **Limitation:** Validated in lung cancer patients, lacking disease-specific items and EC-specific surgical and treatment risks; **Applicability:** Basic framework for initial admission screening of EC patients, requiring supplementation of gynecological oncology-related items.
Early Screen for Discharge Planning, ESDP (73)	At Admission	Cancer patients (74)	Non-EC patients	4/-	Age, Living Alone Status, Disability Status, Self-reported Walking Limitations	**Strength:** Concise and efficient (4 items), quickly identifying patients needing complex discharge plans; **Limitation:** Basic dimensions, lacking gynecological cancer-specific assessment and comprehensive information for detailed plan formulation; **Applicability:** Rapid triage of EC patients on admission to screen high-risk individuals requiring multidisciplinary team intervention.
Inpatient-to-outpatient discharge handoff tool (75)	Prior to Discharge	Patients with Hematologic Malignancies (76)	Non-EC patients	-/6	primary care provider and hospital providers, discharge date and diagnosis, follow-up appointments, pending labs/tests/procedures, other pertinent information, and home care services arranged	**Strength:** Focuses on accurate information transfer among nursing teams, ensuring continuous care; **Limitation:** Highly dependent on systematic implementation and lacks disease-specific items; **Applicability:** Standardized template for EC patients' discharge handover to prevent key information omission.
B. Tools validated in surgical/general inpatient populations (with potential applicability to EC)						
Readiness for Hospital Discharge Scale, RHDS (69)	Prior to Discharge	Patients Undergoing Enterostomy (77) and surgical patients (78)	Non-EC patients	4/21	Personal Status, Knowledge Status, Adaptive Capacity, Anticipated Support	**Strength:** Comprehensive assessment of discharge readiness (including subjective feelings), a gold-standard tool; **Limitation:** Lacks evaluation of cancer, gynecological surgery-specific issues, and items like sexual function and lymphedema; **Applicability:** Basic tool for assessing discharge readiness of EC surgical patients, supplemented by EC-specific interviews recommended.
Patient–reported readiness for hospital discharge, PT-RHDS) (79, 80)	Prior to Discharge	Orthopedic patients (81)	Non-EC patients	4/21	Personal Status, Knowledge, Coping Ability, and Expected Support	**Strength:** Patient-reported, directly reflecting self-perception and needs; **Limitation:** Lacks cancer-specific content (same as RHDS), including gynecological cancer-specific items; **Applicability:** Core tool for EC patients' pre-discharge self-assessment to empower patients and identify potential issues.
Nurse Pre-discharge Checklist (17)	Prior to Discharge	Patients with acute cardiovascular diseases (17)	Non-EC patients	-/15	Confirm Discharge Details,Patient Transport,Post-Acute Care,Medical Supplies,Home Care,Medical Record,Patient Education,Follow-Up Appointments,Prescribed Plans,Understanding Check,Emergency Contacts,Discharge Summary,Signature Collection,Documentation	**Strength:** Detailed content covering all discharge tasks; **Limitation:** Designed for cardiovascular diseases, it lacks items related to sexual function and lymphedema; **Applicability:** Usable as a nursing checklist after significant customization based on EC surgical pathways.
Re-Engineered Discharge (RED) (82)	During Hospitalization	Vascular surgery patients (83)	Non-EC patients	-/12	Health status, Medicines, Appointments, Home services, and Plan for what to do if a problem arises	**Strength:** Structured multi-component process program, proven to reduce readmission rates; **Limitation:** Lacks gynecological cancer-specific content and requires sufficient institutional resources and coordination capacity; **Applicability:** Core components serve as a blueprint for optimizing EC patients' discharge processes.
The martin postoperative discharge screening tool, MPOST) (84)	Prior to Discharge	Surgical intensive care unit (85)	Non-EC patients	-/7	patient age, respiratory rate, atrial Fibrillation, poor renal function, amount of urea nitrogen in the blood, blood glucose level, levels of serum chloride	**Strength:** Based on objective physiological data, effectively predicts post-discharge outcomes of non-critically ill surgical patients; **Limitation:** Relies solely on physiological indicators, ignoring psychosocial, behavioral factors and items on sexual function/lymphedema; **Applicability:** Objective screening tool for medical safety of EC patients before discharge, requiring combination with psychosocial assessment tools.
C. Tools validated in general internal medicine/geriatric populations (with limited applicability)						
LACE index (86)	Prior to Discharge	Heart failure (87)	Used in EC patients	4/-	Length of stay, Acuity, Comorbidity, Emergency department visits	**Strength:** 4 administrative/diagnostic data points, concise and automatable for readmission risk prediction; **Limitation:** Narrow dimensions (lacks sexual function/lymphedema items), unable to guide individualized discharge interventions; **Applicability:** Trend prediction and stratified management of EC patients' readmission risk at the hospital level, not for personalized care plans.
HOSPITAL Score (88)	Prior to Discharge	Pediatric inpatients (89)	Non-EC patients	-/7	Discharge Hemoglobin < 12 g/dL (120 g/L), Oncology Discharge, Discharge Serum Sodium < 135 mEq/L, Surgery During Admission, Non-elective Admission (e.g., Urgent/Emergency), Hospitalizations in Prior Year, Length of Stay ≥ 5 Days	**Strength:** Similar to LACE, identifies high-risk patients via objective data; **Limitation:** Developed for pediatrics, it has no sexual function/lymphedema items, and some indicators may overestimate risks in post-EC surgical patients; **Applicability:** Uncertain predictive validity in EC population, not recommended as first choice, requiring further validation.
Nursing Needs Assessment Instrument, NNAI) (90)	During Hospitalization	Adult inpatients (90)	Non-EC patients	——	cognitive/behavioral/emotional status, health status, functional status, finances, environmental factors in postdischarge care, anticipated skilled care requirements for discharge, meeting continuing care needs	**Strength:** Wide-ranging dimensions, covering overlooked factors like post-discharge environment and economy; **Limitation:** General content (no sexual function/lymphedema items), lacking depth for cancer patients' acute/recovery phase-specific needs; **Applicability:** Framework referenceable, usable for EC patients after integrating oncology nursing expertise.
The 8Ps Risk Assessment Tool (91)	During Hospitalization	——	Non-EC patients	8/-	Problem medications Polypharmacy Psychological conditions Principal diagnosis Poor health literacy Patient support Prior hospitalizations Palliative care	**Strength:** Includes palliative care, a critical dimension for advanced cancer patients; **Limitation:** Developed for general internal medicine (no sexual function/lymphedema items), not optimized for surgical recovery pathways; **Applicability:** Some dimensions are referenceable for EC patients, serving as a supplementary screening tool.
IDEAL Discharge Planning Overview, Process, and Checklist) (92, 93)	Entire Hospitalization Period (Admission to Follow-up)	Geriatric patients (94)	Non-EC patients	——	Include, Discuss, Educate, Assess, and Listen	**Strength:** Emphasizes patient/family-centered communication and participation, an excellent process principle; **Limitation:** Macro framework (no sexual function/lymphedema items) without quantifiable assessment results; **Applicability:** Integrate core concepts into EC patients' discharge preparation process to improve experience.

The symbol "—" denotes either the absence of a unified consensus or the lack of available data.

(1) Strength: Refers to the advantages of the tool when applied to EC surgical patients; (2) Limitation: Refers to the constraints of the tool when applied to EC surgical patients; (3) Applicability: Refers to the description of the scope of use and application value of the tool in the EC surgical patient population.

For example, tools derived from general oncological populations (Part A), such as the Caregiver Preparedness Scale (CPS) and the Quality of Discharge Teaching Scale (ODTS), show reasonable content validity for broad cancer care issues. However, their structural validity within the EC subpopulation remains unverified. Instruments widely recognized as gold standards in surgical settings, such as the Readiness for Hospital Discharge Scale (RHDS) and its patient version (PT-RHDS) (Part B), exhibit strong reliability and construct validity in general surgical cohorts. Nonetheless, their ecological and content validity are limited for EC patients due to the exclusion of disease-specific sequelae, such as those related to gynecological cancer. Predictive tools such as the LACE index (Part C), which excel in criterion and predictive validity for readmission risk, are primarily based on administrative data and lack the ability to address subjective patient readiness or guide individualized discharge planning.

In summary, the current psychometric evidence for discharge readiness tools remains fragmented. Instruments validated for general populations lack EC-specific content validity, while predictive tools fail to provide actionable insights for personalized care planning. Addressing these gaps requires the development of an EC-specific discharge readiness tool that integrates key domains such as sexual health and lymphedema management within a psychometrically sound framework, such as the Readiness for Hospital Discharge Scale (RHDS). Future research should focus on rigorous cross-cultural adaptation and validation to ensure applicability across diverse patient populations.

## 4 Constructing discharge readiness service content for EC patients

The development of discharge readiness services for EC requires the establishment of a specialized framework that is centered on the characteristics of the disease. This framework should integrate aspects of oncologic treatment, such as the extent of surgery and the need for hormone therapy, as well as patient metabolic profiles, including the high prevalence of comorbidities like obesity, and long-term survivorship management needs. The content construction should adhere to two fundamental mechanisms: a multidimensional needs assessment and an interdisciplinary collaboration model.

### 4.1 Scientific multidimensional needs assessment mechanism

While traditional needs assessments relying on caregiver reports are limited by subjectivity, evidence demonstrates that structured, objective tools can significantly improve patient outcomes. For instance, the implementation of standardized risk assessment tools has been associated with a 12-30% reduction in avoidable hospital readmissions in general surgical populations by enabling early identification of high-risk patients (21, 22). Furthermore, integrating patient-reported outcome measures (PROMs) into routine assessments has been shown to significantly improve patient-provider communication and identify unmet needs in domains like psychological distress and physical function, leading to more tailored interventions and enhanced quality of life (23). To advance this field in EC care, future research should leverage multi-source data integration, including machine learning models trained on specific clinical datasets (24), to develop predictive, multidimensional assessment systems. Such a system would synthesize physiological status (e.g., recovery biomarkers), psychological status (e.g., anxiety/depression), social support, and environmental factors. Crucially, the transition from theoretical models like QCNN_BaOpt (24) to clinical impact requires validation in EC cohorts, with a focus on quantifying their efficacy in improving concrete endpoints such as readmission rates, patient satisfaction, and symptom burden.

### 4.2 Systematic interdisciplinary collaboration model

The collaboration within a multidisciplinary team (MDT) is fundamental to delivering high-quality discharge readiness services, with robust evidence supporting its impact on patient outcomes. The discharge team for EC should comprise essential members, including gynecologic oncologists, surgeons, nurses, rehabilitation therapists, nutritionists, psychologists/psychiatrists, and social workers (25, 26). The success of MDT collaboration is contingent upon clearly defined roles, effective communication strategies, and shared decision-making processes. Quantitative analyses demonstrate that structured MDT care can lead to a significant reduction in unplanned 30-day readmissions for complex chronic conditions by improving care coordination and patient education. Specifically, relevant studies (27, 28) have confirmed that the reduction rate can reach 7.4% in direct comparison (from 16.4% to 9.0%) and even exceed 6 percentage points in sustained practice (from 18.9% to 12.6%). Evidence suggests that implementing structured collaboration models, such as the relationship-centered care model, can mitigate team conflicts and further contribute to these positive outcomes (25). Programs like the ASSIST discharge initiative, which extend collaboration to community providers and actively involve patients and caregivers (29, 30), are critical. This collaborative framework, which encompasses the entire continuum from hospitalization to post-discharge follow-up, is essential for ensuring that medical care plans are effectively aligned with patients’ needs at home, ultimately translating into quantifiable improvements in readmission rates and quality of life.

### 4.3 Nurse-led personalized service implementation

Nurses, as pivotal coordinators and implementers of discharge readiness services, play a crucial role in optimizing postoperative outcomes for surgical patients. Meta-analyses of adult surgical inpatients have demonstrated that nurse-led discharge interventions significantly reduce 30-day readmissions and emergency department visits, while simultaneously improving patients’ activities of daily living, quality of life, and overall satisfaction (31). The effectiveness of these interventions lies in their ability to facilitate more coordinated and comprehensible care transitions, aligning with evidence that nurse-driven strategies outperform conventional approaches in minimizing unplanned healthcare utilization and enhancing patient-reported outcomes within surgical populations.

In the early recovery phase, evidence-based health education has emerged as a cornerstone for empowering patient self-management (32). Specifically, a review focusing on ERAS in minimally invasive gynecologic oncology surgery confirmed that nurse-led discharge education, as a key component of ERAS, increases rates of same-day discharge without elevating readmission risk, while also reducing perioperative and postoperative opioid use without compromising pain control (33). Research underscores the importance of personalized education plans grounded in the best available evidence and tailored to address critical postoperative needs, including activity guidance, treatment adherence, and comorbidity management. To uphold patient-centered care principles, it is essential to adopt structured communication techniques such as the teach-back method, which not only ensures comprehension but also actively engages patients and their caregivers in collaboratively developing individualized discharge plans. Evidence further indicates that inadequate patient engagement is strongly associated with an elevated risk of readmission (34). To ensure continuity of care beyond hospital discharge, implementing a dynamic and iterative cycle of “assessment, education, feedback, and adjustment” is paramount. This framework supports seamless transitions from hospital to home, enabling nurses to provide adaptive, ongoing care that meets patients’ evolving needs during postoperative recovery.

## 5 Implementation of discharge readiness services for EC patients

The implementation strategy for discharge readiness services exerts a direct and measurable impact on patient outcomes, with variations in effectiveness observed across different care models, such as the case management model and the primary nursing model. Evidence from a study involving total joint arthroplasty patients demonstrated that the perioperative surgical case management model was associated with a significantly reduced 30-day unplanned readmission rate of 2.1%, compared to the typical 5%-10% reported with standard nursing care. Although specific data on length of hospital stay were not provided, refined perioperative interventions under this model may contribute to a slight increase in hospitalization duration (35). This trade-off underscores the necessity for patient-specific strategy optimization, balancing the benefits of reduced readmissions with potential extensions in hospital stay. Patient-specific factors, such as low Activities of Daily Living (ADL) scores (≤60), the need for tube feeding, poorly managed comorbidities, and limited capacity of primary caregivers, have been identified as significant predictors of extended hospital stays (36). These factors highlight the critical importance of tailoring discharge readiness strategies to the individual circumstances and risk profiles of each patient. Systematic reviews evaluating interventions such as care coordination, discharge education, and follow-up provide mixed results (37), further emphasizing the need for personalized approaches that consider both the clinical and psychosocial aspects of postoperative recovery. In light of these findings, developing and implementing discharge readiness models that integrate individualized risk assessments and patient-centered interventions is crucial for improving outcomes. Future research should prioritize identifying predictive factors and refining care strategies to enable a seamless transition from hospital to home, ultimately enhancing recovery while minimizing unnecessary healthcare utilization.

Advancements in contemporary information technology offer innovative opportunities to enhance discharge readiness services, particularly through the integration of digital health solutions. Digital health platforms, encompassing telemedicine applications and online resource portals, have demonstrated their capacity to strengthen communication between healthcare providers and patients, facilitate access to offline services, and support the collection of real-world post-discharge data for service optimization (38, 39). A meta-analysis of 24 randomized controlled trials (RCTs) involving 9,634 patients with heart failure revealed that eHealth self-management interventions—a critical subset of digital health technologies—significantly reduced all-cause and heart failure-related 30-day readmission rates. These interventions also increased medication adherence by 82% and improved self-care behaviors, outcomes primarily driven by enhanced patient engagement (40). These findings underscore the transformative potential of digital health in fostering better health outcomes through patient-centered approaches.

Moreover, machine learning technologies are emerging as powerful tools for analyzing complex clinical datasets, identifying risk patterns, and predicting individual patient needs. Mobile health (mHealth) and AI predictive models are increasingly being integrated into the postoperative transitional care of gynecological cancer patients. Bukke (41) highlighted that AI-driven mobile remote monitoring can continuously track vital signs and preemptively alert medical teams to complications like lymphedema and thrombosis. The “Zhi Kang Fu” WeChat mini-program, developed and implemented in a top-tier hospital in Beijing, China, by Zhang (42), consolidates six core modules, including health education, complication warnings, chemotherapy management, and psychological support. Patients can access personalized care suggestions via intelligent inquiries, and a 1-month trial showed high user acceptance (156.15 ± 30.93 out of a possible 175), significantly enhancing self-management and adherence during home recovery. Delanerolle further confirmed that mHealth platforms powered by machine learning can provide real-time risk assessments of postoperative symptoms and send reminders to both patients and healthcare providers, ensuring a seamless transition from hospital to home care (43). Khudhur also emphasized that the combination of AI predictive models and mobile technology can detect recurrences or treatment-related complications early, offering continuous, precise, and cost-effective support for gynecological cancer patients outside the hospital setting (44).

By harnessing these capabilities, personalized discharge plans and follow-up strategies can be developed to address the unique requirements of diverse patient populations (45). For instance, studies have demonstrated the utility of neural network models in predicting risks associated with discharge delays or optimizing discharge decisions for specific clinical conditions, such as COVID-19 (46, 47). While promising, the application, effectiveness, and generalizability of these technologies within emergency care and oncology-specific discharge readiness services require further investigation and rigorous validation. In summary, digital health platforms and machine learning methodologies hold significant potential to revolutionize discharge readiness strategies. Future research should focus on validating these technologies in diverse clinical settings, including oncology, to ensure their reliability, scalability, and ability to deliver personalized, data-driven care transitions.

Developing a standardized discharge readiness pathway for esophageal cancer patients requires a comprehensive approach that spans the entire care continuum, from hospital admission to post-discharge recovery. Key strategies for pathway implementation include: ① Tiered health education tailored to the disease stage, cognitive function, and self-management capabilities of each patient.② Diverse educational formats, such as in-person instruction, personalized educational materials, audiovisual aids, and digital platforms, to accommodate varying patient preferences and learning needs.③ Structured communication techniques, including the teach-back method, which has proven effective in enhancing patient comprehension, engagement, and knowledge retention (48). A standardized pathway that integrates approaches such as staff training in teach-back techniques, discharge resource folders, and optimized educational documents has demonstrated a synergistic effect. Compared to *ad-hoc* discharge practices, these strategies significantly improve patients’ satisfaction with discharge-related services. Evidence from satisfaction surveys reveals notable improvements in care transition scores (from 52.41% to 54.49%) and discharge information scores (from 87.38% to 90.12%) (49). These findings highlight the value of a structured, patient-centered discharge readiness pathway in achieving better patient-reported outcomes. Furthermore, the integration of technological tools with humanistic and patient-centered care is essential for optimizing resource allocation and providing continuous support during recovery (13, 50). By facilitating seamless communication and personalized interventions, these tools complement the standardized pathway and contribute to improved postoperative outcomes for EC patients.

### 5.1 Core steps of the EC surgery patient discharge readiness service implementation flowchart

**Figure 1:**
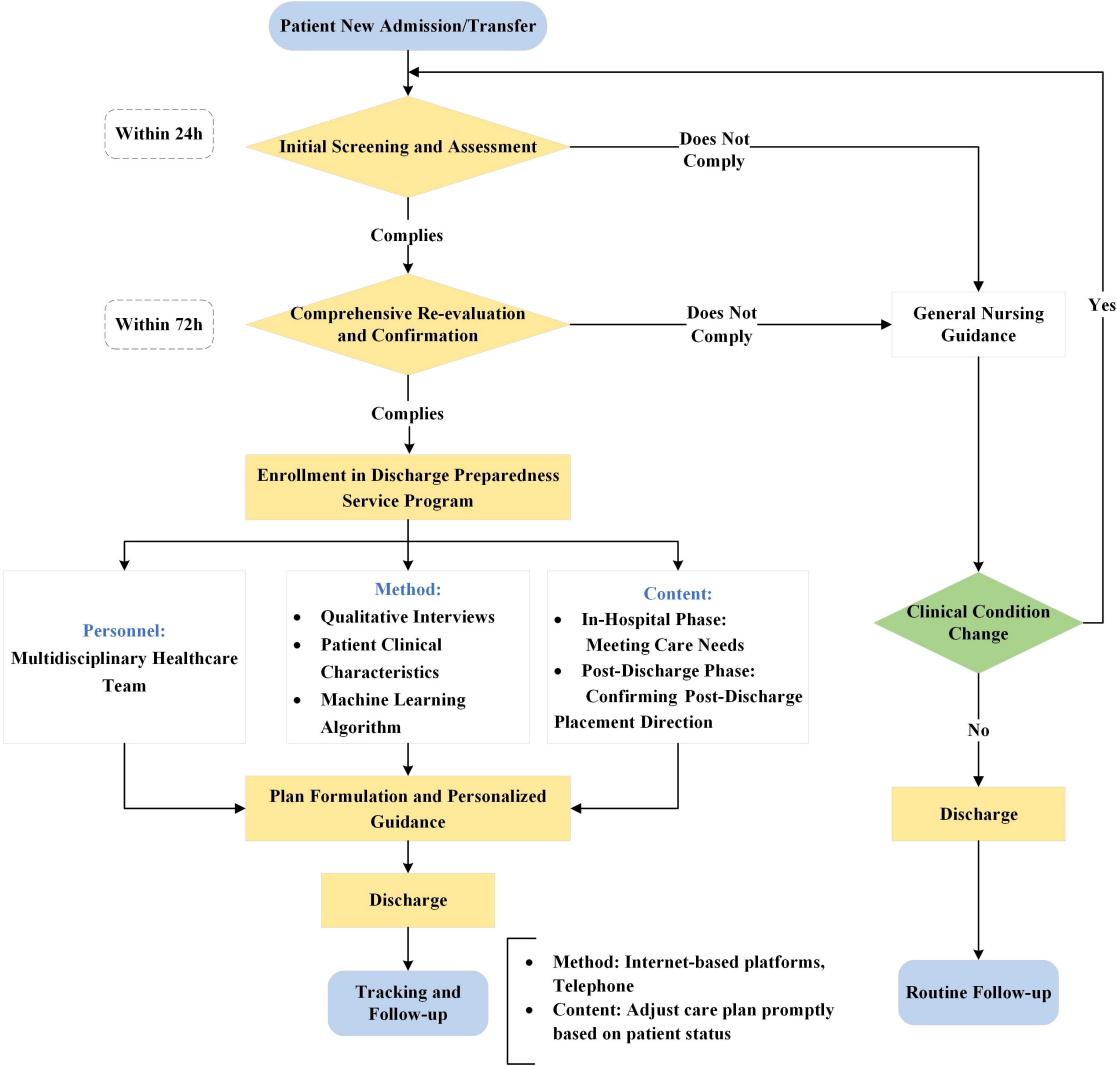
Implementation flowchart for EC discharge readiness services.

The core implementation process of the EC surgery patient discharge readiness service is visualized in a detailed flowchart (**Figure 1**), which consists of four sequential and interconnected phases as follows.

#### 5.1.1 Screening and initial assessment

Within 24 hours of patient admission or transfer, the healthcare team conducts an initial screening using the Blaylock Risk Assessment Screening Score (BRASS) (51)to identify patients at risk for delayed discharge or those with complex care needs. A total score of 10 or higher indicates the need to proceed to the next step (52, 53).

#### 5.1.2 Comprehensive reassessment and confirmation

For patients who screen positive, a comprehensive assessment should be conducted within 72 hours (50). The assessment should cover domains such as Activities of Daily Living (ADLs), self-care ability and need for assistance, transportation arrangements, home support network, medication management ability, nutritional status and eating ability, and home care service needs (e.g., home visit nursing, hospice care). The use of tools like the Comprehensive Discharge Planning Needs Assessment Form is recommended (54, 55).

#### 5.1.3 Plan development and personalized guidance

Following the reassessment outcomes, MDT collaboratively formulates a personalized discharge plan. The implementation of this plan is structured in phases:①Inpatient Phase: Address in-hospital care requirements and provide training for patients and caregivers on essential post-discharge care skills. ②Discharge Phase: Confirm the discharge destination and coordinate subsequent care arrangements. ③Home Care Phase: Equip primary caregivers with necessary skills and offer information on home nursing services and community resource linkages. ④Transfer to Facility: Contact and coordinate with receiving institutions to ensure a smooth transition.

#### 5.1.4 Tracking and follow-up

Subsequent to discharge, scheduled follow-ups via telephone, home visits, or clinic appointments should be conducted to evaluate recovery progress, care satisfaction, and any unmet needs, allowing for necessary adjustments to the care plan (56). The frequency and mode of follow-up should be tailored to the individual patient’s condition and risk assessment.

## 6 Outcome evaluation of discharge readiness services for EC patients

Assessing the effectiveness of discharge readiness services is crucial for the ongoing enhancement of healthcare quality. An effective evaluation system should incorporate clinical evidence alongside multidimensional indicators. Research indicates that among patients undergoing minimally invasive hysterectomy, there is no significant difference in the 60-day complication rates between obese and non-obese patients (5.1% vs. 5.4%, P = 0.86), although obese patients experience lower rates of same-day discharge (57). A recent large-scale analysis of the National Cancer Database found that for patients with Type II endometrial cancer undergoing robot-assisted laparoscopy, the 30-day readmission rate was 2.5%, showing no significant difference compared to conventional laparoscopy (2.4%, p=0.819) (58). Factors such as extended hospital stays (>1 day vs. ≤1 day: 40.0% vs. 23.0%, P = 0.04) and postoperative complications (63.3% vs. 13.4%, P<0.01) are significant predictors of readmission (59). The implementation of Enhanced Recovery After Surgery (ERAS) protocols has been shown to effectively reduce hospital stays and associated costs without significantly increasing readmission rates (60). These findings offer a foundation for optimizing discharge strategies tailored to various surgical techniques and patient demographics.

Building upon prognostic evidence and established guideline requirements, such as those outlined in the 2022 ESMO Clinical Practice Guideline for endometrial cancer (32), the evaluation of outcomes necessitates a comprehensive framework that integrates patient outcomes, caregiver experiences, and the utilization of healthcare system resources. The MDT should develop personalized, risk-stratified follow-up plans that consider recurrence risk, treatment side effects, physical and mental health status, and resource accessibility. According to the ESMO guideline, follow-up frequency should be stratified by risk: for low-risk patients, clinical visits are recommended every 6 months for the first 2 years and then annually until 5 years, with telephone follow−up as an acceptable alternative; for high-risk patients, visits are recommended every 3 months for the first 3 years and then every 6 months until 5 years. Patient education on symptom recognition and the option of patient−initiated follow−up (PIFU) are also emphasized (32). Short-term evaluations should concentrate on critical clinical indicators, such as 30-day postoperative complication rates, readmission rates, and the time required for bowel function recovery (61). In contrast, long-term evaluations should incorporate Patient-Reported Outcomes (PROs), employing standardized instruments like the Health Education Impact Questionnaire (HeiQ) to assess self-management domains, including health behaviors, emotional well-being, and social interaction (62). Recent studies have further demonstrated the value of the EORTC QLQ-EN24—an endometrial-cancer-specific PRO module—in capturing post-discharge quality of life. Sobočan et al. applied the Slovenian QLQ-EN24 to 79 EC survivors and found that gastrointestinal symptoms, musculoskeletal pain and sexual/vaginal concerns were significantly linked to poorer multi-domain outcomes, underscoring the scale’s sensitivity to long-term sequelae (63). Likewise, the prospective QLEC cohort (n = 198) administered the QLQ-EN24 together with PROMIS measures and showed that pre-operative obesity predicted worse physical functioning, incident lower-extremity lymphedema and diminished sexual interest, providing baseline risk data for discharge planning (64). In Taiwan, Li et al. validated the Chinese QLQ-EN24 in 105 women ≥ 6 months post-treatment and revealed that those with lower-limb lymphedema reported significantly more severe climacteric symptoms and poorer physical, role, emotional, cognitive and social functioning, confirming the instrument’s cross-cultural utility (65). Integrating this EC-specific tool into routine follow-up therefore enables clinicians to detect symptom clusters, functional deficits and psychosocial needs after hospital discharge, informing tailored self-management support. This internationally validated scale is instrumental in identifying deficiencies in post-discharge self-management, thereby informing interventions for continuity of care. The establishment of a continuous improvement cycle—comprising evidence generation, outcome evaluation, and service optimization—facilitates the enhancement of quality throughout the patient management journey in EC.

## 7 Conclusion

This review systematically synthesizes existing evidence on discharge readiness services for patients undergoing EC surgery, highlighting its essential role in facilitating a seamless transition from hospital to home and enhancing long-term outcomes. The key findings are as follows (1): The variability of assessment tools necessitates adaptation to the specific characteristics of EC; (2) The effectiveness of service implementation is contingent upon the synergy of multidisciplinary collaboration, nurse-led education (e.g., the teach-back method), and the integration of technology (e.g., remote monitoring digital platforms); (3) Systems for evaluating outcomes must incorporate both clinical indicators (e.g., 30-day readmission rates) and patient-reported outcomes (e.g., the Health Education Impact Questionnaire [HeiQ] scale) to comprehensively assess multidimensional recovery status. Persistent challenges include the lack of standardized pathways, fragmented community resources, and insufficient patient engagement. To address these issues, we propose the following at the policy level: the development of guideline-based and culturally adapted standardized discharge pathways. At the research level, conducting prospective trials to compare various implementation models is essential for advancing evidence-based nursing management practices. For future prospective trials, the proposed flowchart can serve as the Standard Operating Procedure for the intervention group. This approach ensures that the complex intervention—the Discharge Preparedness Service Program—is delivered in a standardized and systematic manner, minimizing potential bias arising from inconsistent interpretations of the intervention by different researchers. The flowchart outlines clear operational details, including specified time points (e.g., initial screening within 24 hours, comprehensive re-evaluation within 72 hours), designated personnel (a multidisciplinary healthcare team), core methods (integration of qualitative interviews and machine learning algorithms), and intervention content (assessment of both in-hospital and post-discharge patient needs). By providing a detailed and structured blueprint, this flowchart significantly enhances the reproducibility of the trial and improves the comparability of results across studies. Moreover, the flowchart inherently highlights critical processes and outcome measures requiring attention in future trials. This ensures that subsequent studies maintain a consistent focus on evaluating the efficacy of the intervention and its impact on patient outcomes, thereby strengthening the evidence base for nursing management strategies.

The flowchart provided (**Figure 1**) serves as a practical and actionable framework for clinicians, highlighting the necessity for local adaptation in light of disparities in healthcare resource allocation. Future research should aim to advance evidence-based standardized practices while maintaining flexibility rooted in patient-centered principles.

## 8 Limitations

This study has several limitations. First, as a narrative review, its methodology inherently has inherent limitations. We did not conduct a systematic and exhaustive literature search, nor did we use standardized quality assessment tools to perform rigorous risk of bias assessment on all included studies. Literature screening and inclusion relied more on the researchers’ subjective judgments and considerations regarding the representativeness and heuristic value of the content. This may introduce selection bias and undermine the robustness of the conclusions. Second, the literature search was restricted to Chinese and English databases, excluding gray literature and studies in other languages. This may limit the comprehensiveness of the evidence obtained. Therefore, the conclusions of this paper are intended to provide a comprehensive overview and practical framework for this field, and its viewpoints and recommendations need to be verified and supported by more rigorous systematic reviews, meta-analyses, or original studies in the future.

## References

[1] Sung H Ferlay J Siegel RL Laversanne M Soerjomataram I Jemal A . Global cancer statistics 2020: GLOBOCAN estimates of incidence and mortality worldwide for 36 cancers in 185 countries. CA Cancer J Clin. (2021) 71:209–49. doi: 10.3322/caac.21660, PMID: 33538338

[2] Chinese Anti-Cancer Association GOC . Guidelines for diagnosis and treatment of endometrial carcinoma (2021 edition). China Oncol. (2021) 31:501–12. doi: 10.19401/j.cnki.1007-3639.2021.06.08

[3] Zhang X Wang JD . Research progress on epidemiological risk factors related to endometrial cancer. Med Recapitulate. (2021) 27:2995. doi: 10.3969/j.issn.1006-2084.2021.15.016

[4] Zhou S Qin YZ Zhang L Zhang HQ Liu X Jiang JF . Status and influencing factors of body image in gynecological cancer patients receiving postoperative chemotherapy. Nurs Res China. (2021) 35:306. doi: 10.12102/j.issn.1009-6493.2021.02.022

[5] Jiang Z . Incidence and influencing factors of postoperative lower limb lymphedema in cervical cancer patients. Modern Nurse. (2021) 28:55. doi: 10.19791/j.cnki.1006-6411.2021.25.017

[6] Xia LL Dai LN Deng C . Application of preoperative prehabilitation nursing model based on ERAS concept in gynecological tumor patients. China Modern Med. (2024) 31:162. doi: 10.3969/j.issn.1674-4721.2024.02.039

[7] Wang L Xu L . Role of reducing average length of stay in improving hospital operational efficiency. China Health Standard Management. (2017) 8:17. doi: 10.3969/j.issn.1674-9316.2017.12.010

[8] Mäkelä P Stott D Godfrey M Ellis G Schiff R Shepperd S . The work of older people and their informal caregivers in managing an acute health event in a hospital at home or hospital inpatient setting. Age Ageing. (2020) 49:856–64. doi: 10.1093/ageing/afaa085, PMID: 32428202 PMC7444665

[9] Landeiro F Roberts K Gray AM Leal J . Delayed hospital discharges of older patients: A systematic review on prevalence and costs. Gerontologist. (2019) 59:e86–97. doi: 10.1093/geront/gnx028, PMID: 28535285

[10] Newton C Nordin A Rolland P Ind T Larsen-Disney P Martin-Hirsch P . British Gynaecological Cancer Society recommendations and guidance on patient-initiated follow-up (PIFU). Int J Gynecol Cancer. (2020) 30:695–700. doi: 10.1136/ijgc-2019-001176, PMID: 32312719

[11] Fenwick AM . An interdisciplinary tool for assessing patients’ readiness for discharge in the rehabilitation setting. J Adv Nurs. (1979) 4:9–21. doi: 10.1111/j.1365-2648.1979.tb02984.x, PMID: 254690

[12] Weiss ME Piacentine LB . Psychometric properties of the readiness for hospital discharge scale. J Nurs Meas. (2006) 14:163–80. doi: 10.1891/jnm-v14i3a002, PMID: 17278337

[13] Galvin EC Wills T Coffey A . Readiness for hospital discharge: A concept analysis. J Adv Nurs. (2017) 73:2547–57. doi: 10.1111/jan.13324, PMID: 28440958

[14] Naylor MD Aiken LH Kurtzman ET Olds DM Hirschman KB . The care span: The importance of transitional care in achieving health reform. Health Aff (Millwood). (2011) 30:746–54. doi: 10.1377/hlthaff.2011.0041, PMID: 21471497

[15] Weiss ME Bobay KL Bahr SJ Costa L Hughes RG Holland DE . A model for hospital discharge preparation: from case management to care transition. J Nurs Adm. (2015) 45:606–14. doi: 10.1097/nna.0000000000000273, PMID: 26502068

[16] Coleman EA Parry C Chalmers S Min SJ . The care transitions intervention: results of a randomized controlled trial. Arch Intern Med. (2006) 166:1822–8. doi: 10.1001/archinte.166.17.1822, PMID: 17000937

[17] Mennuni M Gulizia MM Alunni G Francesco Amico A Maria Bovenzi F Caporale R . ANMCO Position Paper: hospital discharge planning: recommendations and standards. Eur Heart J Suppl. (2017) 19:D244–d55. doi: 10.1093/eurheartj/sux011, PMID: 28751845 PMC5526471

[18] Yam CH Wong EL Cheung AW Chan FW Wong FY Yeoh EK . Framework and components for effective discharge planning system: a Delphi methodology. BMC Health Serv Res. (2012) 12:396. doi: 10.1186/1472-6963-12-396, PMID: 23151173 PMC3508885

[19] Mui J Cheng E Salindera S . Enhanced recovery after surgery for oncological breast surgery reduces length of stay in a resource limited setting. ANZ J Surg. (2024) 94:1096–101. doi: 10.1111/ans.18901, PMID: 38488251

[20] Ortiz Vazquez EF Londoño Victoria JC Castillo López GA Hidalgo Tapia EC Mena Acosta FI Puchaicela Namcela SDR . Minimally invasive gynecologic surgery and enhanced recovery and outcomes: A literature review. Cureus. (2025) 17:e84814. doi: 10.7759/cureus.84814, PMID: 40568263 PMC12188281

[21] Bertsimas D Li ML Paschalidis IC Wang T . Prescriptive analytics for reducing 30-day hospital readmissions after general surgery. PloS One. (2020) 15:e0238118. doi: 10.1371/journal.pone.0238118, PMID: 32903282 PMC7480861

[22] Pham H Hitos K Pawaskar R Sinclair JL Mathuthu H Nahm CB . Multidisciplinary protocol to reduce surgical readmissions in Australia: American College of Surgeons National Surgical Quality Improvement Program. ANZ J Surg. (2025) 95:335–41. doi: 10.1111/ans.19252, PMID: 39431747

[23] Gibbons C Porter I Gonçalves-Bradley DC Stoilov S Ricci-Cabello I Tsangaris E . Routine provision of feedback from patient-reported outcome measurements to healthcare providers and patients in clinical practice. Cochrane Database Syst Rev. (2021) 10:Cd011589. doi: 10.1002/14651858.CD011589.pub2, PMID: 34637526 PMC8509115

[24] Nandhini RS Lakshmanan R . QCNN_BaOpt: multi-dimensional data-based traffic-volume prediction in cyber-physical systems. Sensors (Basel). (2023) 23:1485. doi: 10.3390/s23031485, PMID: 36772525 PMC9919015

[25] Gaboury I Lapierre LM Boon H Moher D . Interprofessional collaboration within integrative healthcare clinics through the lens of the relationship-centered care model. J Interprof Care. (2011) 25:124–30. doi: 10.3109/13561820.2010.523654, PMID: 21182442

[26] Irwin RS Flaherty HM French CT Cody S Chandler MW Connolly A . Interdisciplinary collaboration: the slogan that must be achieved for models of delivering critical care to be successful. Chest. (2012) 142:1611–9. doi: 10.1378/chest.12-1844, PMID: 23208334

[27] Hahn B Ball T Diab W Choi C Bleau H Flynn A . Utilization of a multidisciplinary hospital-based approach to reduce readmission rates. SAGE Open Med. (2024) 12:20503121241226591. doi: 10.1177/20503121241226591, PMID: 38249952 PMC10798118

[28] Patel H Yirdaw E Yu A Slater L Perica K Pierce RG . Improving early discharge using a team-based structure for discharge multidisciplinary rounds. Prof Case Manage. (2019) 24:83–9. doi: 10.1097/ncm.0000000000000318, PMID: 30688821

[29] Kraun L De Vliegher K Vandamme M Holtzheimer E Ellen M van Achterberg T . Older peoples’ and informal caregivers’ experiences, views, and needs in transitional care decision-making: a systematic review. Int J Nurs Stud. (2022) 134:104303. doi: 10.1016/j.ijnurstu.2022.104303, PMID: 35797843

[30] Better together – ASSIST hospital discharge scheme. Nottinghamshire, UK: Mansfield District Council (MDC) (2016). Available online at: https://www.nice.org.uk/sharedlearning/better-together-assist-hospital-discharge-scheme-ahds (Accessed March 2025).

[31] Mao H Xie Y Shen Y Wang M Luo Y . Effectiveness of nurse-led discharge service on adult surgical inpatients: A meta-analysis of randomized controlled trials. Nurs Open. (2022) 9:2250–62. doi: 10.1002/nop2.1268, PMID: 35661429 PMC9374412

[32] Oaknin A Bosse TJ Creutzberg CL Giornelli G Harter P Joly F . Endometrial cancer: ESMO Clinical Practice Guideline for diagnosis, treatment and follow-up. Ann Oncol. (2022) 33:860–77. doi: 10.1016/j.annonc.2022.05.009, PMID: 35690222

[33] Aubrey C Nelson G . Enhanced recovery after surgery (ERAS) for minimally invasive gynecologic oncology surgery: A review. Curr Oncol. (2023) 30:9357–66. doi: 10.3390/curroncol30100677, PMID: 37887577 PMC10605820

[34] Lin L Fang Y Wei Y Huang F Zheng J Xiao H . The effects of a nurse-led discharge planning on the health outcomes of colorectal cancer patients with stomas: A randomized controlled trial. Int J Nurs Stud. (2024) 155:104769. doi: 10.1016/j.ijnurstu.2024.104769, PMID: 38676992

[35] Alem N Rinehart J Lee B Merrill D Sobhanie S Ahn K . A case management report: a collaborative perioperative surgical home paradigm and the reduction of total joint arthroplasty readmissions. Perioper Med (Lond). (2016) 5:27. doi: 10.1186/s13741-016-0051-2, PMID: 27777752 PMC5067901

[36] Po HW Chu YC Tsai HC Chen CY Chiu YW . Evaluate the differential effectiveness of the case management and primary nursing models in the implementation of discharge planning. J Clin Nurs. (2024) 34:3753–3775. doi: 10.1111/jocn.17550, PMID: 39528412

[37] Jesus TS Stern BZ Lee D Zhang M Struhar J Heinemann AW . Systematic review of contemporary interventions for improving discharge support and transitions of care from the patient experience perspective. PloS One. (2024) 19:e0299176. doi: 10.1371/journal.pone.0299176, PMID: 38771768 PMC11108181

[38] Optimal care pathway for women with endometrial cancer. Australia: Cancer Council Victoria and Department of Health Victoria (2021). Available online at: https://www.cancer.org.au/ (Accessed March 2025).

[39] Couturier B Carrat F Hejblum G . Comparing patients’ Opinions on the hospital discharge process collected with a self-reported questionnaire completed via the internet or through a telephone survey: an ancillary study of the SENTIPAT randomized controlled trial. J Med Internet Res. (2015) 17:e158. doi: 10.2196/jmir.4379, PMID: 26109261 PMC4526961

[40] Liu S Li J Wan DY Li R Qu Z Hu Y . Effectiveness of eHealth self-management interventions in patients with heart failure: systematic review and meta-analysis. J Med Internet Res. (2022) 24:e38697. doi: 10.2196/38697, PMID: 36155484 PMC9555330

[41] Bukke SPN Kumarachari RK Rajasekhar ESK Dudekula JB Kamati M . Computational intelligence techniques for achieving sustainable development goals in female cancer care. Discover Sustainability. (2024) 5:390. doi: 10.1007/s43621-024-00575-x

[42] Zhang K Zhang Y Xiao Q . Development and usability evaluation of a WeChat mini program for home management of patients with gynecological Malignancies. Chin Nurs Management. (2024) 24:1256–60. doi: 10.3969/j.issn.1672-1756.2024.08.014

[43] Delanerolle G Jouaiti M Pathiraja V Mudalige T Eleje GU Mbwele B . A rapid scoping review on the use of artificial intelligence applications in women’s health (MARIE WP1). doi: 10.20944/preprints202503.1920.v1

[44] Khudhur YS . Artificial intelligence in obstetrics and gynecology: Current applications and future perspectives. Int J Obstetrics Gynaecology. (2025). doi: 10.33545/26648334.2025.v7.i1a.36

[45] Hu HT Hong SS Jia YY Song JP . Advances in application of machine learning in discharge readiness services. Chin J Nursing. (2024) 59:378. doi: 10.3761/j.issn.0254-1769.2024.03.018

[46] Meng Q Liu W Gao P Zhang J Sun A Ding J . Novel deep learning technique used in management and discharge of hospitalized patients with COVID-19 in China. Ther Clin Risk Manage. (2020) 16:1195–201. doi: 10.2147/tcrm.S280726, PMID: 33324064 PMC7733409

[47] Safavi KC Khaniyev T Copenhaver M Seelen M Zenteno Langle AC Zanger J . Development and validation of a machine learning model to aid discharge processes for inpatient surgical care. JAMA Netw Open. (2019) 2:e1917221. doi: 10.1001/jamanetworkopen.2019.17221, PMID: 31825503 PMC6991195

[48] Oh EG Lee HJ Yang YL Kim YM . Effectiveness of discharge education with the teach-back method on 30-day readmission: A systematic review. J Patient Saf. (2021) 17:305–10. doi: 10.1097/pts.0000000000000596, PMID: 30882616

[49] Thum A Ackermann L Edger MB Riggio J . Improving the discharge experience of hospital patients through standard tools and methods of education. J Healthc Qual. (2022) 44:113–21. doi: 10.1097/jhq.0000000000000325, PMID: 35231013

[50] Zurlo A Zuliani G . Management of care transition and hospital discharge. Aging Clin Exp Res. (2018) 30:263–70. doi: 10.1007/s40520-017-0885-6, PMID: 29313293

[51] Ma MZ Fan YY Yang PP Du X B Li Y . Translation and application of blaylock risk assessment screening scale. Nurs Res China. (2022) 36:1901. doi: 10.12102/j.issn.1009-6493.2022.11.004

[52] Louis Simonet M Kossovsky MP Chopard P Sigaud P Perneger TV Gaspoz JM . A predictive score to identify hospitalized patients’ risk of discharge to a post-acute care facility. BMC Health Serv Res. (2008) 8:154. doi: 10.1186/1472-6963-8-154, PMID: 18647410 PMC2492858

[53] Leong MQ Lim CW Lai YF . Comparison of Hospital-at-Home models: a systematic review of reviews. BMJ Open. (2021) 11:e043285. doi: 10.1136/bmjopen-2020-043285, PMID: 33514582 PMC7849878

[54] Stoller JK . COPD exacerbations: Prognosis, discharge planning, and prevention. Available online at: https://sso.uptodate.com/contents/zh-Hans/copd-exacerbations-prognosis-discharge-planning-and-prevention?search=COPD%20exacerbations%3A%20Prognosis%2C%20discharge%20planning%2C%20and%20prevention&source=search_result&selectedTitle=1%7E150&usage_type=default&display_rank=1 (Accessed February 1, 2025).

[55] Wang H Wang Y Liu Y Ying SY Le X Ke J . Construction of core practice indicator system and related forms for discharge planning. Nurs Res China. (2022) 36:189. doi: 10.12102/j.issn.1009-6493.2022.02.001

[56] Alper E O’Malley TA Greenwald J . Discharge and readmission. Available online at: https://sso.uptodate.com/contents/zh-Hans/hospital-discharge-and-readmission?search=%E5%87%BA%E9%99%A2%E4%B8%8E%E5%86%8D%E5%85%A5%E9%99%A2&source=search_result&selectedTitle=1%7E150&usage_type=default&display_rank=1 (Accessed March 8, 2025).

[57] Burdette ER Pelletier A Flores MN Hinchcliff EM St Laurent JD Feltmate CM . Same-day discharge after minimally invasive hysterectomy for endometrial cancer and endometrial intraepithelial neoplasia in patients with morbid obesity: Safety and potential barriers. Int J Gynecol Cancer. (2025) 35:100042. doi: 10.1016/j.ijgc.2024.100042, PMID: 39878291

[58] Lamiman K Silver M Goncalves N Kim M Alagkiozidis I . Impact of robotic assistance on minimally invasive surgery for type II endometrial cancer: A national cancer database analysis. Cancers. (2024) 16:9. doi: 10.3390/cancers16142584, PMID: 39061223 PMC11274470

[59] Liang MI Rosen MA Rath KS Clements AE Backes FJ Eisenhauer EL . Reducing readmissions after robotic surgical management of endometrial cancer: a potential for improved quality care. Gynecol Oncol. (2013) 131:508–11. doi: 10.1016/j.ygyno.2013.09.033, PMID: 24096114

[60] Mendivil AA Busch JR Richards DC Vittori H Goldstein BH . The impact of an enhanced recovery after surgery program on patients treated for gynecologic cancer in the community hospital setting. Int J Gynecol Cancer. (2018) 28:581–5. doi: 10.1097/igc.0000000000001198, PMID: 29466256

[61] Chau JPC Liu X Lo SHS Chien WT Hui SK Choi KC . Perioperative enhanced recovery programmes for women with gynaecological cancers. Cochrane Database Syst Rev. (2022) 3:Cd008239. doi: 10.1002/14651858.CD008239.pub5, PMID: 35289396 PMC8922407

[62] Skorstad M de Rooij BH Jeppesen MM Bergholdt SH Ezendam NPM Bohlin T . Self-management and adherence to recommended follow-up after gynaecological cancer: results from the international InCHARGE study. Int J Gynecol Cancer. (2021) 31:1106–15. doi: 10.1136/ijgc-2020-002377, PMID: 33858949

[63] Sobočan M Gašpar D Gjuras E Knez J . Evaluation of patient-reported symptoms and functioning after treatment for endometrial cancer. Curr Oncol. (2022) 29:5213–22. doi: 10.3390/curroncol29080414, PMID: 35892983 PMC9394308

[64] Warring S Yost KJ Cheville AL Dowdy SC Faubion SS Kumar A . The quality of life after endometrial cancer study: baseline characteristics and patient-reported outcomes. Curr Oncol. (2024) 31:5557–72. doi: 10.3390/curroncol31090412, PMID: 39330040 PMC11431380

[65] Li CC Chang TC Chang CW Huang CH Tsai YF Huang CL . Quality of life and climacteric symptoms in women with endometrial cancer: examining the impact of lower limb lymphedema. J Patient Rep Outcomes. (2025) 9:66. doi: 10.1186/s41687-025-00895-0, PMID: 40512268 PMC12165921

[66] Archbold PG Stewart BJ Greenlick MR Harvath T . Mutuality and preparedness as predictors of caregiver role strain. Res Nurs Health. (1990) 13:375–84. doi: 10.1002/nur.4770130605, PMID: 2270302

[67] Henriksson A Andershed B Benzein E Arestedt K . Adaptation and psychometric evaluation of the Preparedness for Caregiving Scale, Caregiver Competence Scale and Rewards of Caregiving Scale in a sample of Swedish family members of patients with life-threatening illness. Palliat Med. (2012) 26:930–8. doi: 10.1177/0269216311419987, PMID: 21908520

[68] Hauksdóttir A Valdimarsdóttir U Fürst CJ Onelöv E Steineck G . Health care-related predictors of husbands’ preparedness for the death of a wife to cancer--a population-based follow-up. Ann Oncol. (2010) 21:354–61. doi: 10.1093/annonc/mdp313, PMID: 19633052

[69] Weiss ME Piacentine LB Lokken L Ancona J Archer J Gresser S . Perceived readiness for hospital discharge in adult medical-surgical patients. Clin Nurse Spec. (2007) 21:31–42. doi: 10.1097/00002800-200701000-00008, PMID: 17213738

[70] Yang MM Liang W Zhao HH Zhang Y . Quality analysis of discharge instruction among 602 hospitalized patients in China: a multicenter, cross-sectional study. BMC Health Serv Res. (2020) 20:647. doi: 10.1186/s12913-020-05518-6, PMID: 32652990 PMC7353724

[71] Blaylock A Cason CL . Discharge planning predicting patients’ needs. J Gerontol Nurs. (1992) 18:5–10. doi: 10.3928/0098-9134-19920701-05, PMID: 1629535

[72] Leonetti A Peroni M Agnetti V Pratticò F Manini M Acunzo A . Thirty-day mortality in hospitalised patients with lung cancer: incidence and predictors. BMJ Support Palliat Care. (2023) 14:e2003–e2010. doi: 10.1136/spcare-2023-004558, PMID: 37666650

[73] Holland DE Harris MR Leibson CL Pankratz VS Krichbaum KE . Development and validation of a screen for specialized discharge planning services. Nurs Res. (2006) 55:62–71. doi: 10.1097/00006199-200601000-00008, PMID: 16439930

[74] Socwell CP Bucci L Patchell S Kotowicz E Edbrooke L Pope R . Utility of Mayo Clinic’s early screen for discharge planning tool for predicting patient length of stay, discharge destination, and readmission risk in an inpatient oncology cohort. Support Care Cancer. (2018) 26:3843–9. doi: 10.1007/s00520-018-4252-8, PMID: 29777381

[75] Moy NY Lee SJ Chan T Grovey B Boscardin WJ Gonzales R . Development and sustainability of an inpatient-to-outpatient discharge handoff tool: a quality improvement project. Jt Comm J Qual Patient Saf. (2014) 40:219–27. doi: 10.1016/s1553-7250(14)40029-1, PMID: 24919253

[76] Prince M Allen D Chittenden S Misuraca J Hockenberry MJ . Improving transitional care: the role of handoffs and discharge checklists in hematologic Malignancies. Clin J Oncol Nurs. (2019) 23:36–42. doi: 10.1188/19.Cjon.36-42, PMID: 30681999

[77] Li S Luo C Xie M Lai J Qiu H Xu L . Factors influencing readiness for hospital discharge among patients undergoing enterostomy: A descriptive, cross-sectional study. Adv Skin Wound Care. (2024) 37:319–27. doi: 10.1097/asw.0000000000000159, PMID: 38767424

[78] Nurhayati N Songwathana P Vachprasit R . Surgical patients’ experiences of readiness for hospital discharge and perceived quality of discharge teaching in acute care hospitals. J Clin Nurs. (2019) 28:1728–36. doi: 10.1111/jocn.14764, PMID: 30589480

[79] Weiss M Yakusheva O Bobay K . Nurse and patient perceptions of discharge readiness in relation to postdischarge utilization. Med Care. (2010) 48:482–6. doi: 10.1097/MLR.0b013e3181d5feae, PMID: 20393364

[80] Bobay KL Weiss ME Oswald D Yakusheva O . Validation of the registered nurse assessment of readiness for hospital discharge scale. Nurs Res. (2018) 67:305–13. doi: 10.1097/nnr.0000000000000293, PMID: 29877987

[81] Kamau EB Foronda C Hernandez VH Walters BA . Reducing length of stay and hospital readmission for orthopedic patients: A quality improvement project. J Dr Nurs Pract. (2021). doi: 10.1891/jdnp-d-20-00060, PMID: 34716277

[82] Re-Engineered Discharge(RED) . Toolkit: agency for healthcare research and quality. Available online at: https://www.ahrq.gov/hai/red/toolkit/postdischarge-doc.html (Accessed March 19, 2025).

[83] Vogel TR Kruse RL Schlesselman C Doss E Camazine M Popejoy LL . A qualitative study evaluating the discharge process for vascular surgery patients to identify significant themes for organizational improvement. Vascular. (2024) 32:395–406. doi: 10.1177/17085381221135267, PMID: 36287544

[84] Parker C Griffith DH . Reducing hospital readmissions of postoperative patients with the martin postoperative discharge screening tool. J Nurs Adm. (2013) 43:184–6. doi: 10.1097/NNA.0b013e3182895902, PMID: 23528682

[85] Toich L . Novel tool could predict hospital readmissions (2016). Available online at: https://www.pharmacytimes.com/view/novel-tool-could-predict-hospital-readmissions (Accessed April 1, 2025).

[86] van Walraven C Dhalla IA Bell C Etchells E Stiell IG Zarnke K . Derivation and validation of an index to predict early death or unplanned readmission after discharge from hospital to the community. Cmaj. (2010) 182:551–7. doi: 10.1503/cmaj.091117, PMID: 20194559 PMC2845681

[87] Wang H Robinson RD Johnson C Zenarosa NR Jayswal RD Keithley J . Using the LACE index to predict hospital readmissions in congestive heart failure patients. BMC Cardiovasc Disord. (2014) 14:97. doi: 10.1186/1471-2261-14-97, PMID: 25099997 PMC4128541

[88] Donzé JD Williams MV Robinson EJ Zimlichman E Aujesky D Vasilevskis EE . International validity of the HOSPITAL score to predict 30-day potentially avoidable hospital readmissions. JAMA Intern Med. (2016) 176:496–502. doi: 10.1001/jamainternmed.2015.8462, PMID: 26954698 PMC5070968

[89] da Silva NC Albertini MK Backes AR das Graças Pena G . Validation of the HOSPITAL score as predictor of 30-day potentially avoidable readmissions in pediatric hospitalized population: retrospective cohort study. Eur J Pediatr. (2023) 182:1579–85. doi: 10.1007/s00431-022-04795-z, PMID: 36693994

[90] Holland DE Hansen DC Matt-Hensrud NN Severson MA Wenninger CR . Continuity of care: a nursing needs assessment instrument. Geriatr Nurs. (1998) 19:331–4. doi: 10.1016/s0197-4572(98)90119-7, PMID: 9919118

[91] Kim CS Flanders SA . In the clinic. Transitions of care. Ann Intern Med. (2013) 158:Itc3–1. doi: 10.7326/0003-4819-158-5-201303050-01003, PMID: 23460071

[92] Strategy 4: care transitions from hospital to home: IDEAL discharge planning. US: Agency for Healthcare Research and Quality (2013). Available online at: https://www.ahrq.gov/professionals/systems/hospital/engagingfamilies/strategy4/index.html (Accessed April 2025).

[93] Luther B Wilson RD Kranz C Krahulec M . Discharge processes: what evidence tells us is most effective. Orthop Nurs. (2019) 38:328–33. doi: 10.1097/nor.0000000000000601, PMID: 31568123

[94] Williams A Kesten KS . Engaging older adults and families using the IDEAL discharge protocol: A quality improvement initiative to improve outcomes and reduce readmissions. J Gerontol Nurs. (2023) 49:13–9. doi: 10.3928/00989134-20230915-04, PMID: 37768584

